# The impact of unplanned 30-day readmission as a quality indicator in pediatric surgery

**DOI:** 10.3389/fsurg.2023.1199659

**Published:** 2023-05-31

**Authors:** Sarah Ellul, Mohamed Shoukry

**Affiliations:** ^1^ Division of Paediatric Surgery, Department of Surgery, Mater Dei Hospital, Swatar, Malta; ^2^ Division of Paediatric surgery, Consultant Paediatric and Neonatal Surgeon, Department of Surgery, Mater Dei Hospital, Swatar, Malta

**Keywords:** readmission rate, financial burden, post-operation, strategies, challenge

## Abstract

**Introduction:**

Hospital readmission is one of the indicators used to assess quality of service provided in healthcare. Based on accumulated knowledge, risk management teams assess data related to readmissions to find curative solutions for underlying factors. The current article's aim is investigating readmission routes within the workplace in paediatric surgery service during the first 30 days post discharge from Mater Dei Hospital (MDH).

**Materials and method:**

A retrospective study of children's hospital readmissions between October 2017 and November 2019 was performed, strictly before COVID-19 pandemic. Demographics and clinical records including age, gender, pre-existing comorbidities, diagnosis during primary admission and readmission, procedure carried out, ASA grade, length of stay, and outcomes were collected. All children re-admitted under a single paediatric surgical department within 30 days from initial admission to tertiary referral hospital were included. Patients undergoing emergency visitation without subsequent admissions were excluded. Readmissions were classified into cohorts: elective and emergency, depending on the nature of primary admission. Contributing factors and outcomes were compared.

**Results:**

935 surgical admissions (221 elective and 714 emergencies) were registered at MDH over the given period, with an average hospital stay of 3.62 days. Total readmission rate was 1.7% (*n* = 16). 25% (*n* = 4) of readmissions were post elective, 75% (*n* = 12) post emergency admission, with an average stay of 4.37 days and no mortalities. 43.7% (*n* = 7) were re-admissions post-surgical intervention. Further surgical interventions were necessary in 25% (*n* = 4) of readmitted patients, the remainder (*n* = 12) treated conservatively.

**Conclusion:**

Published reports concerning paediatric surgical readmission rates are limited, challenging healthcare systems. Most readmissions area voidable; therefore, healthcare workers must provide adequate strategies tailored to their resources, efficient multidisciplinary approaches with improved communication to decrease morbidity and prevent readmissions.

## Introduction

More frequently, physicians are being asked to provide evidence for measurably high quality and patient safety. Measurements of length of hospital stays, post-operative complications and readmission rates are being used more frequently as hospital quality indicators (Isobel H. Marks, 2016).

Avoidable hospital readmissions are a vital topic of research of national focus since these can be potential indicators of unnecessary expenditure and clinical failure (C. Feudtner, 2009).

Unplanned surgical readmissions can lead to a significant increase in healthcare workloads, amounting to serious financial burden, whilst also negatively effecting patients' experience and outcomes. Paediatric unplanned hospital readmissions were estimated to cost the healthcare system in USA, UK and Canada up to approximately $1billion per annum. (Wijlaars, 2016), (C. Feudtner, 2009), (J. G. Berry, 2013).

In view of these implications on patient outcomes and healthcare costs, readmissions are increasingly being monitored as a measure of hospital and surgeon quality and performance (Afif N. Kulaylat, 2017).

Existing international literature on hospital variations on unplanned paediatric readmission is relatively quite limited, mostly this has been noted to be related to the difficulty in tracking readmissions across different hospitals and over time (Naomi. S. Bardach, 2013).

In view of this, the topic of readmission has become an important issue for doctors and hospital management since unplanned surgical readmissions to hospitals have been noted to play a key role as indicators of quality-of-care measurement throughout the healthcare services (Bardach N. S., 2013).

Readmission rates following surgical procedures can vary considerably, with multiple factors being accountable for this matter. Some of these actors are thought to be associated with patient level related factors example: age and pre-operative medical conditions whilst other factors include: surgeon and hospital- level factors (G. Spolverato, 2014) (O., 2013) (D. P. Richardson, 2013).

Establishing the specific risk factors which could potentially be associated with unplanned readmissions is a key determinant to assist rate reduction. In a systematic review conducted in 2011, multiple risk factors were identified. These included: medical comorbidities, length of stay and previous hospital admissions (D. Kansagara, 2011).

Interestingly, whilst most of the studies related to hospital readmissions have mostly focused on the adult populations, research studies on the pediatric population have been mostly limited to general pediatric conditions or single-institution series.

Our aim was to investigate the readmission workload within the first 30 days post discharge from hospital including current process, identifying any potential contributing factors which could be linked to unplanned readmissions and the impact and outcomes in the setting of a paediatric surgical practice at Mater Dei Hospital (MDH).

## Materials and methods

### Data and population

A retrospective study of the paediatric surgical patients which were re-admitted to Mater Dei Hospital was performed between October 2017 and November 2019. All the patients involved underwent an elective or/and emergency admission within the previous 30 days. The search was based on the availability of patients' hospital notes and admission records.

Local institutional authority approval was granted by the Mater Dei Hospital Data Protection Board in January 2020, prior to the initiation of the data collection. No approval from the research committee was required as advised by the national data protection unit.

Demographic details collected include age, gender, pre-existing comorbidities, initial diagnosis during the primary admission and procedure carried out, ASA (American Society of Anesthesiologists) and its outcomes, including treatment and length of stay, as well as the diagnosis for the second readmission and outcomes. The thirty-day readmission was calculated in relation to the original initial day of admission and all re-admissions within 30 days were examined. This thirty-day readmission was defined as any unplanned admissions, with an overnight stay, within 30 days of any elective or emergency hospital episode.

Inclusion criteria included all paediatric surgical patients (< 14 years of age) which were re-admitted under a single paediatric surgical department within 30 days from their initial admission for both elective (including day case surgeries) and emergency admissions at Mater Dei Hospital, between October 2017 and November 2019. Those patients who underwent any planned re-admission, an accident and emergency visitation without any subsequent admission to hospital, or any re-admissions under a different specialty. Or any patients with missing data were excluded from the study.

Only patients < 14 years of age were included in this study since the current protocol in our local hospital states that any patient above 14 years of age, should be treated by the adult surgical team.

Readmissions were later classified into two different cohorts: elective and emergency cohorts, depending on the nature of the primary admission that is whether they were initially admitted for an elective procedure or else admitted in view of an acute problem *via* the Accident and Emergency Department. Contributing factors and outcomes for both these groups were then compared.

### Statistical analysis

Continuous data that were not normally distributed were expressed as median, Standard Deviation (SD) and interquartile ranges (IQR), and categorical variables as figures and percentages. Other complex analysis was not carried out in view of the small cohort of patients.

### Outcomes

Our primary outcome of interest was the total paediatric surgical 30-day unplanned readmission rates over a specific time frame, whilst the secondary outcomes included: the length of hospital stay, morbidity rates throughout the 30-day period and the evaluation of factors associated with unplanned readmissions.

## Results

### Demographics

A total of 985 paediatric surgical admissions were identified among 3 years at Mater Dei Hospital from a paediatric population of approximately 78,000 in the Maltese islands (1), from which 935 patients met all inclusion criteria, with an age range of 1 month to 13 years and an overall female to male ratio of 0.7:1. Patient characteristics are presented in Table 1.

**Table 1:** Patient characteristics for both Emergency and elective admissions.

	Emergency	Elective
Age		
**<1yr**	192	70
**1**–**5 years**	140	71
**5**–**10years**	186	65
**10**–**14 years**	196	15
Sex		
**Male**	393	157
**Female**	321	64
Race		
**Caucasian**	680	199
**Non-Caucasian**	34	22
Co-morbidities		
**Yes**	38	15
**No**	676	206

Elective admissions accounted for 24% (*n* = 221) of admissions, whilst 76% (*n* = 714) were emergency admissions. There were 78 patients admitted in 2017, 5.13% (*n* = 4) elective admissions and 94.8% (*n* = 74) emergency admissions. In 2018, there were 516 admissions, 76% (*n* = 393) were emergency admission, whilst 24% (*n* = 123) were elective admissions. Between January 2019 and November 2019, a total of 341 admissions were registered. 72% (*n* = 247) of which were emergency admissions, whilst 28% (*n* = 94) were elective surgical cases (Table 2).

**Table 2:** Cases done between October 2017-October 2019.

	2017	2018	2019
Emergency	74	393	247
Elective	4	123	94

From the total admissions, 24% (*n* = 221) were elective admission listed for elective surgical operations, whereas the remaining 76% (*n* = 714) were emergency surgical admissions admitted *via* the Accident and Emergency.

From October 2017 until end of December 2017 a total of 78 patients were admitted. From these 5.13% (*n* = 4) were elective admissions, which underwent elective surgical operations and 94.8% (*n* = 74) were emergency admissions.

**Figure 1:**
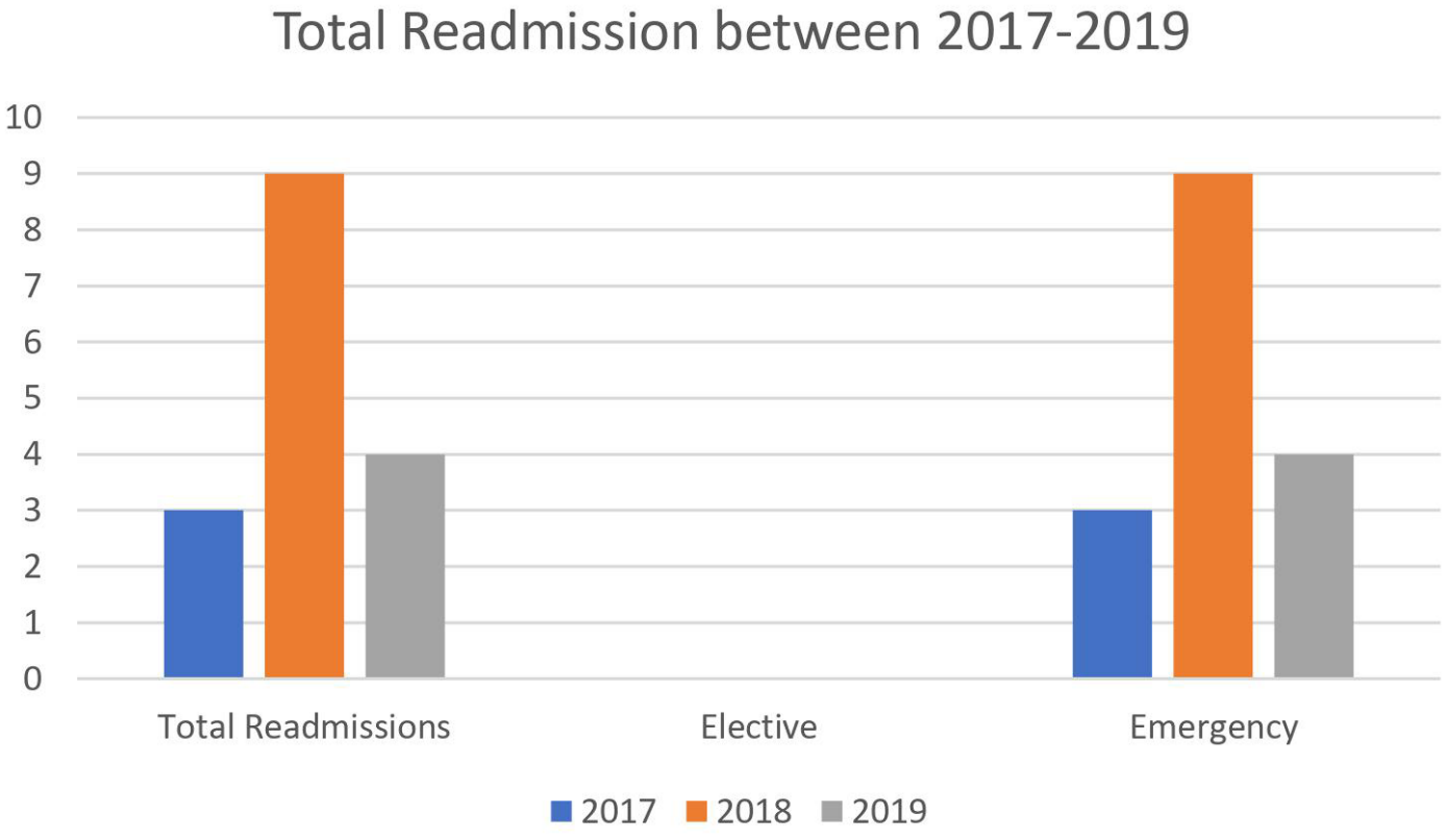
Total admissions between 2017 and 2019.

During 2019, 72% (*n* = 247) of all admissions were emergency cases, whilst 28% (*n* = 94) were elective surgical admissions. All this can be depicted in Figure 1.

A general review on gender admissions throughout the 3-year period revealed no statistical significance of note and therefore the results were not included in the study.

### Median Age for admission and length of hospital stay

There was minimal change in the median age for admissions for both elective and emergency cases between this study period. In 2017, the median age for elective and emergency admissions was 5 years. For 2018, the median age for emergency admissions was 5 years and that of elective admissions was 4 years, whilst for 2019, the median age for the emergency admissions was found to be 6 year and that of the elective admissions was 4 years. The median age for the emergency admissions was found to be 6 years and that of the elective admissions was 4 years.

The length of hospital stay was also compared. In 2017, the average length of stay was 1.8 days. In 2018, the average length of stay was 1.49 days for the emergency admissions, with a standard deviation (SD) of 6.17, whilst the average length of stay for the elective admissions was 1.03 days, with a SD of 1.32. In 2019, the average length of stay for the emergency cases was 2.45 days and that of elective admissions was found to be 1.44 days.

### Indications for admission

Elective admissions carried out included weekly admissions for elective surgeries such as day care surgeries and major surgical procedures with a varying amount in their length of stay. The day care cases included cases such as: umbilical and inguinal hernia repairs, orchidopexy and ligation of Processus Vaginalis (excision of hydroceles), excision of skin lesions, cystoscopies and endoscopy procedures including oesophagoduodensocopies (OGD) and colonoscopies. The major surgical procedures included operations such as: pyeloplasty, postero-sagital anorectoplasty (PSARP), insertion of feeding tubes (Gastrostomy/Percutaneous endoscopic gastrostomy (PEG), laparoscopic pyloromyotomy, Nissen fundoplication and laparoscopic pull through. When comparing both types of operations over the mentioned time frame, it was noted that the ratio of day case surgeries: major surgeries was: 18:1. This limited number of major operations was mostly noted to be related to the limited number of major cases made available over the 2-year study period.

### Readmissions

The current study reported a total of 16 patients which have been readmitted within 30 days post physical discharge from hospital. The initial diagnoses, type of initial admission (elective or emergency), surgical action plan, length of stay for both admissions and the interval days between both admissions of all patients were reviewed extensively.

**Figure 2:**
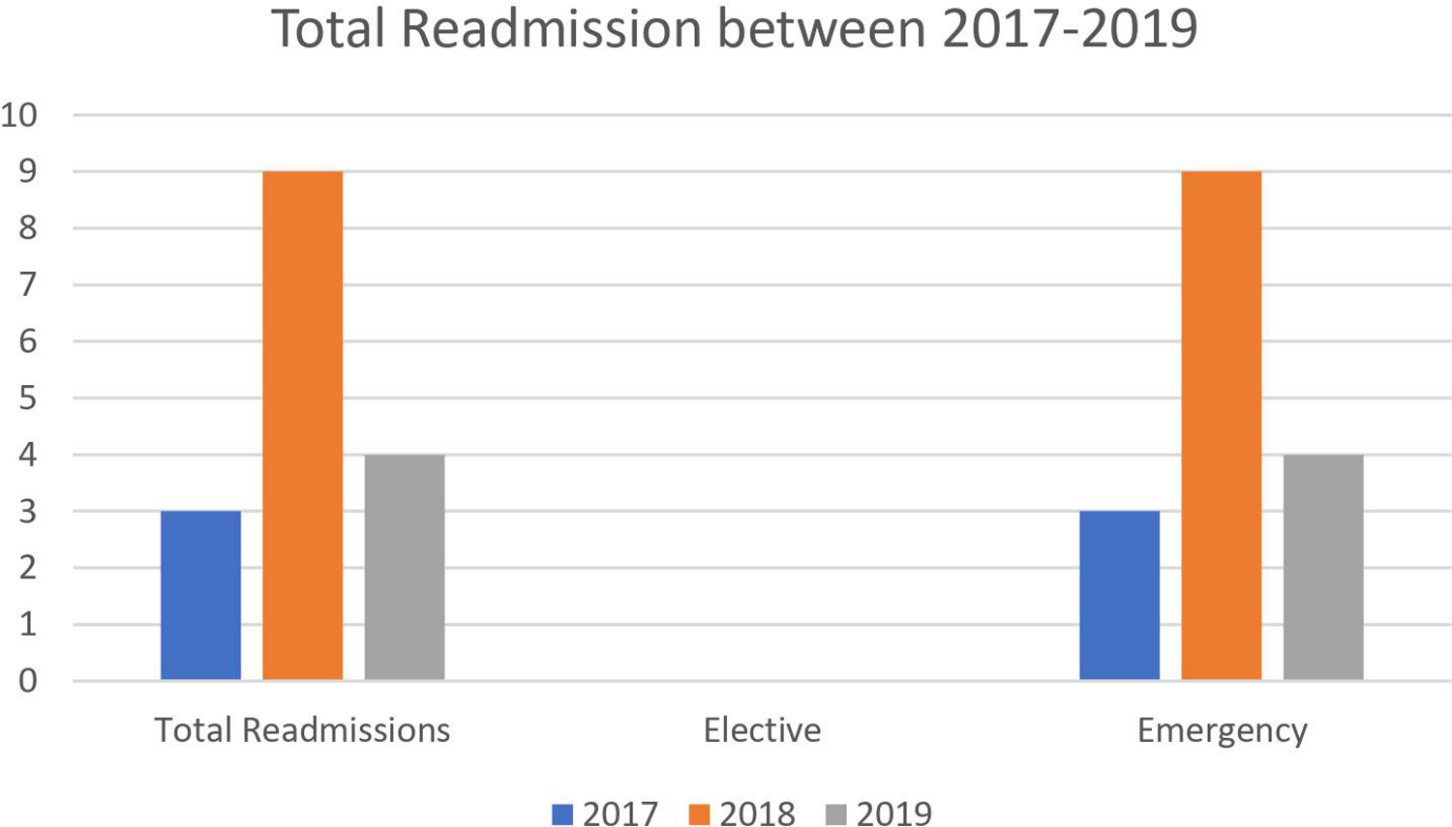
Total readmissions between 2017 and 2019.

The total number of readmissions over this period (25 months), was 1.7% (*n* = 16). From these patients, 1.8% (*n* = 4) were from elective admissions and 16% (*n* = 12) were from emergency admissions. 69% of readmissions (*n* = 11) were male with 31% (*n* = 5) being female. In the current study, males were found to be representing most admissions and are indeed even predominant for readmission (Figure 2).

**Figure 3:**
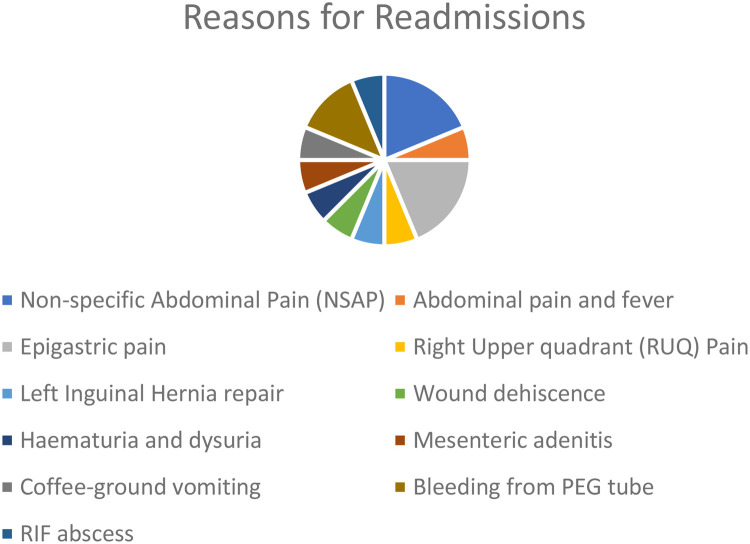
Indications for paediatric surgical patient readmissions.

Overall, the reasons for readmissions included, recurrent non-specific abdominal pain and epigastric pain, bleeding from gastrostomy tube site, hematuria, post-operative wound dehiscence, and right iliac fossa abscess (post a perforated appendicitis) and an incarcerated inguinal hernia (Figure 3).

From the 16 patients which required some form of readmission, 25% (*n* = 4) of these total was post elective admission and the remaining 75% (*n* = 12), were from post emergency admission cohort.

The interquartile range (IQR) for the length of stay during the first admissions of all cases was 3 days. For the elective admissions (*n* = 4) the IQR for length of stay was 2 days and for the emergency admission (*n* = 12) the IQR was 2 days. When looking at their second admission (readmission), the IQR for the length of stay was found to be 8.5 days. The median age for readmissions was 9.6 years (range 3–14 years).

43.7% (*n* = 7) of the total readmissions, were after a surgical intervention. These interventions included: laparoscopic appendicectomy, pyeloplasty, insertion of gastrostomy and inguinal hernia repair.

Further surgical interventions were necessary in 25% (*n* = 4) of the readmitted patients, the remainder (*n* = 12) of which were treated conservatively.

The indication for readmissions and their length of hospital stay can be seen in Table 3.

**Table 3:** Types of readmitted cases and length of hospital stay.

Case Readmissions and length of hospital stay (days)	
Non-specific abdominal pain	5
Epigastric pain	2
Bleeding from gastrostomy site	4
Hematuria	2
Wound dehiscence post-operation	2
Intra-abdominal abscess	13
Incarcerated Hernia	2

The surgical interventions required included: three oesophagoduodensocopies and an inguinal hernia repair for an incarcerated inguinal hernia. Over the 24-month study period, 298 procedures (elective and or emergencies) were undertaken at Mater Dei Hospital, with an overall reoperation rate being 1.34%.

There were no re-admissions which required any radiological intervention on re-admission (Table 4).

**Table 4:** Management of readmissions.

	Management of Readmissions		
	Oct-Dec 2017	Jan-Dec 2018	Jan-Oct 2019
Total	3	9	4
Treated Surgically	0	1	1
Treated Conservatively	3	8	3

None of the patients which were readmitted required any admission to the intensive care unit, and no mortalities were reported from the readmissions documented during this time post-surgical discharge from hospital.

Of all readmissions, 31% (5/16) had some form of pre-morbid medical conditions prior to the initial admissions. These medical conditions included: hypertrophic cardiomyopathy, gastro eosophageal reflux disease, epilepsy, and Cerebral palsy with Christianson syndrome.

From these, only 20% (1/5) required a surgical operation, the rest of which were treated conservatively. This patient was initially admitted in view of gastritis and was managed conservatively. He was later readmitted in view of bleeding from his gastrostomy site, which required a blood transfusion and an OGD to be carried out.

The average interval of days between each admission was 7.25 days (1–25 days). The total unplanned overall readmission rate within the initial 30 days from the first operation was found to be 0.44%.

## Discussion

Understanding unplanned readmissions in the paediatric population provides a crucial opportunity for physicians and surgeons to enhance the delivery and quality of care in paediatric surgical world.

Although previously found to be controversial, it is now well accepted that surgical readmission rates can add up to a tremendous financial burden on the national health care system, leading to a detrimental impact on the patients’ and their families' quality of life. Unexpected readmissions are thought of having significant economic impact, amounting to approximately $17.4 billion per year (2).

In view of this fact, hospital readmission rates are looked upon as potential markers of hospital care quality, with significant penalties issued to those hospitals who do not meet the expected expectations.

Financial competence is critical in the healthcare department, and since most of the surgical readmissions are regarded as preventable readmissions, therefore amenable to a decrease in the health care expenditure, it is regarded as a critical topic. Due to this fact, readmission rates have become a new topic of interest with regards to quality improvement.

*Tsai* et al. noted an interesting relationship between the adherence to the surgical process measures and volume and the procedure specific 30-day risk-adjusted mortality rates, which are all specific measures of the hospital surgical quality (3).

Two important factors which are noticeable in international studies when discussing readmission rates in both adults and paediatric populations is that the working definition for readmission rates varies significantly from one study to another, with no fixed definition present leading to a dramatic difference in readmission rates noticeable between both populations. The lack of standardization on definitions leads to problems when trying to compare data, from different research hospitals, to improve outcomes. Several studies noted that in the paediatric population the unplanned hospital readmission rates range from 3.4% to 28.6% (4–7).

For our local study, we defined any readmission as any in-patient and visits to Accident and Emergency by patients within 30-days following discharge post any paediatric surgical admission (both elective and emergency) leading to an unplanned readmission.

Interestingly, internationally, research delineating paediatric surgical readmissions are quite lacking. Such data is essential since this would help health care workers assess and better understand the reasons for readmissions, whilst also identifying any aspects which could be improved or changed to decrease readmissions.

It was therefore relatively challenging to compare our results to other established data since there is a lack of paediatric surgical studies available (both locally and internationally) for this field. Other limitations related to this study which were present included: the retrospective nature of this study and its small sample size. This has its own limitations, such as bias and confounding, related to imprecisions in data collections.

Additionally, in the surgical readmissions, as opposed to the medical readmissions, the patients which are readmitted rely on the concept that most patients are medically fit prior to their initial admission, therefore this helps in reducing the bias, which is mostly caused through the variation in the patients' presentation or the severity of their illnesses (3). Of note, in a systematic review by Zhou et al, the patients' comorbidity status was found to be a risk factors for unplanned hospital readmission (8).

From our limited local data, we noted that comorbidities and emergent operations were significantly associated with unplanned readmissions.

Locally, 31% of the readmission cohort were found to be suffering from various medical comorbidities. Internationally, large studies of generalized pediatric readmissions had noted that the vast majority of readmissions are associated with complex chronic conditions including neuromuscular, respiratory and cardiovascular conditions that lasted for one year or more or congenital conditions which involved one or more organ systems (Hain P. D., 2013) (Rice-Townsend, 2012) and this was consistent from the results noted from a study by *Burjonrappa et al*. (S. Burjonrappa, 2015).

From our audit, emergency readmissions within the first 30 days of discharge was 0.44% of cases. This value was noted to be less than other previously published overall readmission rates, which also included emergency and elective cases, which had an overall readmission rate of 2% (7) and 4.4% (9). Nonetheless, the current evidence for the readmission rates within the first 30 days in the paediatric surgical practice is still quite limited and ranges from 0.54% to 6.5% (10, 11).

Noticeably, the emergency admissions were associated with a higher readmission rate. 25% (*n* = 4) of the total re-admissions was post elective admissions, which was found to be significantly lower than that of the emergency admission cohort: 75% (*n* = 12).

We managed to compare our results with a local readmission trend analysis carried out by Mater Dei, Clinical Performance Unit, where the local readmission rates of multiple specialties since 2013 were compared. Firstly, we noticed that our results were similar to the data produced by the Clinical Performance Unit, further solidifying our results. Secondly, we were also able to compare our readmission rate to multiple other specialties. What was visible, was that the paediatric surgical readmission rates were one of the lowest readmission rates in Mater Dei when these were compared to both other surgical specialties and medical specialties (12). This should help act as a model in order to further improve patient safety and management.

International adult readmissions rates, which are quoted in multiple literate reviews for general surgery vary considerably, due to its heterogenous properties. A study done by *Jacobs* et al. (13) noted that the overall readmission rate for general surgical patients was of 5.21%, which was at the lower end of the reported readmission rates for France, US, and the UK (14–16).

Of note, the average interval days between initial admission and readmission was 10 days. Interestingly, more than 63% (*n* = 10) of readmissions were readmitted within 10 days from their original discharge. These results are therefore convincing evidence for improvement in the care of these types of patients.

On evaluating the most common reasons for readmission, it was noted that 50% (*n* = 8) of patients readmitted, were readmitted in view of lack of pain management despite being instructed and prescribed analgesia on their original discharge from hospital, whilst also being given a free three-day supply of their medications from hospital. These findings do highlight a potential weakness which our system needs to address and improve on. Perhaps improving the liaison between the hospital setting and the community setting, such as the respective general practitioner, could help us address such an issue better.

Equally important was that 43.7% of our readmissions were found to be post a surgical intervention This could potentially indicate that this is a key factor which could significantly increase readmission rates and careful consideration of such a finding is required.

An important limitation present in our study was the very limited sample size available with a limited time frame period to assess patients and readmission rates. Ideally this data should be reassessed over a longer period to assess whether the same results are applied or not.

An important consideration is that for most of our local discharges, we aim to plan discharge dates well in advance, with most of the patients having a tentative date for discharge on admission. This gives us time to work out any problems which the patients might encounter on discharge and address the issues appropriately. More importantly, the discharge planning is always supervised by senior doctors of the team, whilst ensuring adequate communication and coordination with the nursing team. On discharge, all patients are given a discharge plan, with all plans explained to the parents thoroughly. A copy of the discharge summary is given to the parents as well as leaflets with specific advice related to the presenting complaint or surgical procedure which was carried out. The ward phone number is also available for the parents for them to use as a means of contact with the paediatric surgical team and if needed are also advised to seek immediate surgical care at the Accident and Emergency Department, outside normal working hours.

As previously mentioned, readmissions lead to a significant financial burden on hospitals worldwide. Unfortunately, we were unable to calculate the exact financial burden that our local admissions had on our hospital since this information was not provided by the hospital administration department at the time of writing.An important fact, which can further help reduce readmission rates, is better communications with the community teams outside the hospital, to offer a better multidisciplinary approach for all patients. Equally important, high-risk groups which are deemed of being at risk for potential readmissions should be the focus of quality initiates to minimise any shortcomings.

Ultimately, by identifying the risk factors including patients' characteristics and any events during the hospital stay, which might increase readmission rates, we will create a better transition into the community with a reduction in the early readmissions.

## Conclusion

Overall, hospital readmissions are upsetting for both the patients and their respective families, which also need to adapt to this change in setting. They all represent a dramatic financial cost on the health care system as well as leading to a detrimental effect on the quality of patient care. Importantly, underlying reasons for readmissions in the paediatric surgical populations differ and unplanned readmissions are profusely influenced by patient-related factors and post-operative complications. Therefore, multiple reasons exist as to why health care professionals need to improve their understanding of surgical readmissions.

The current study evaluated readmission rates for paediatric surgical patients whilst also keeping in mind the characteristic features related to the initial mode of presentation and the length of stay of these admissions. The overall readmission rates in this study population are consistently lower than other previously published literature in other countries, yet no other similar studies are available locally to compare the results obtained.

There is limited published data on the readmissions within the initial 30 days from discharge for the paediatric surgical population worldwide. Therefore, it is incredibly difficult to comment on the optimal and fair readmission rate. Noticeably, elective admissions are noted to have a lower readmission rate when compared to emergency admissions, indicating that both the patient and the disease level factors plays an important role in this matter. Ideally, having effective discharge planning as well as coordinated communication between all parties involved, plays a vital role in avoiding unnecessary readmissions.

A great deal of knowledge regarding surgical readmissions already exists, which we have summarized within. However, future efforts should focus on standardizing definitions for readmission and reporting criteria, designing prediction models for surgical patients and ultimately the important task of creating interventions that reduce the morbidity and mortality of these patients and further improve the quality of surgical care. As previously mentioned, these re-admissions are costly, potentially harmful and at times even avoidable. Most surgical readmissions are potentially avoidable, therefore as health care workers we need to provide adequate strategies tailored to our local community to prevent readmissions.

## Data Availability

The raw data supporting the conclusions of this article will be made available by the authors, without undue reservation.
